# Lewis Paul D: R for Medicine and Biology

**DOI:** 10.4103/2153-3539.73505

**Published:** 2010-12-03

**Authors:** G William Moore

**Affiliations:** Pathology and Laboratory Medicine Service, Baltimore VA Maryland Health Care System, Baltimore; Department of Pathology, University of Maryland Medical System; and Department of Pathology, The Johns Hopkins Medical Institutions, Baltimore, USA


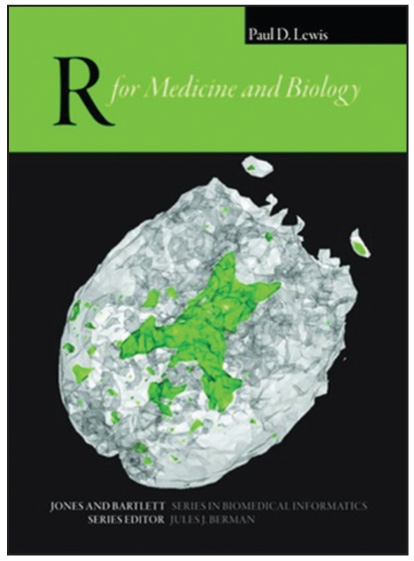
“R” is a freely available, open-source statistical software package, available for Linux, MacOSX^®^, and Microsoft^®^ Windows^®^ operating systems, suitable for statistical analyses and graphical displays of data, with an attached programming language. Historically, R’s commercial predecessor, “S”, was developed at AT&T laboratories in the 1970s, and has become an industry standard among statisticians that can be downloaded from the R project site: http://www.r-project.org/

*R for Medicine and Biology* is a thoughtful and well-written guide for novice users of R. The book highlights biomedical applications, with numerous worked-out sample problems and datasets, as well as program’s source code. The reader is encouraged to execute this code, and introduce one’s own variations. *R for Medicine and Biology* is addressed to students and professionals at all levels in the fields of biology, medicine, and bioinformatics, with an interest in the analysis and visualization of their datasets. While many of the functions of R are duplicated by inexpensive commercial spreadsheets with attached programming languages, such as Microsoft^®^ Excel^®^ combined with Visual Basic^®^ for Applications, R has an integrated programming language, a large range of specialized data analysis methods, and is constantly being updated by a core team and user contributions from the R community.

With R, users may assign values to a single (scalar) variable, vector, matrix, or multidimensional array. Values can assume numeric, logical (true/false), text string, or missing values as data types. Data frames include several different data types. R has functions for descriptive statistics (mean, standard deviation, etc.), vector arithmetic, and sorting. Each R object created in a command line can be printed “on-the-fly”, or a “session” of R-commands can be saved and reloaded at a later time. The user can construct homemade mathematical functions that incorporate arithmetic, logic, and iterative calculations (“while loops”). Chapters in *R for Medicine and Biology* cover sample datasets that might be generated in a biomedical laboratory, such as epidemiologic data, patient records for tumor-node-metastasis (TNM)-staged cancer protocols, tissue microarray datasets, magnetic resonance image (MRI) image-analyses, and genomic databases.

Since many datasets are imported from external sources, R has extensive features for import/export in non-R formats, including traditional comma-separated variable (.csv) records, as well as open-source formats, such as SPSS, Minitab, SAS, Stat, WEKA, and Octave. For larger systems of linked biomedical datasets, the relational database model (RDBM) is the most widely used model for data query and analysis. Cost-free, open-source Structured Query Language (SQL) is used in many academic and government installations. R connects readily to SQL systems, using R-open database connectivity (RODBC) as the application programming interface. *R for Medicine and Biology* provides detailed instructions for connecting R to Microsoft® Access®, a widely used commercial RDBM, as well as to MySQL, a cost-free, open source RDBM.

The two pillars of classical statistics are “estimation” (descriptive statistics) and “hypothesis testing” (inferential statistics). R supports analysis methods in both areas. Descriptive statistics, including mean, median, quartile, range, standard deviation, standard error, etc., provide researchers and managers with a bird’s eye view of selected variables in a dataset. In statistical theory, there is in principle a large, sometimes infinite, “population” of numerical values, described by “parameters”, including population mean, population standard deviation, population median, etc. A given dataset is regarded as a “sample”, drawn from this theoretical population. “Sample statistics”, including sample mean, sample standard deviation, sample median, etc., are calculated as a “least squares best estimate” for theoretical parameters. *R for Medicine and Biology* shows how to calculate these basic parameters in R, and create pie charts, bar charts, dot plots, box-and-whisker plots (box = quartiles, whiskers = range), normal density functions and distributions, and normalized quartiles.

In statistical hypothesis testing, typically there is a “null hypothesis”, or straw man, which asserts that the dataset in question fails to make a diagnosis, fails to predict prognosis, fails to offer safe or effective therapy, etc. The statistical inference question is then whether this dataset is so different from the distribution of datasets predicted by the null hypothesis, that the null hypothesis can be “rejected” at a predetermined “*P* value”. Traditionally, the most popular statistical hypothesis test is the “Student *t*-test”, that compares two samples, either different groups of patients, oligonucleotides, etc. (“unpaired test”), or from “paired” observations such as before and after therapy on the same patient, or match–non-match for a particular oligonucleotide. The *F*-test is a generalization for multiple samples. “Non-parametric” statistical tests are statistical tests that do not assume the normal (Gaussian) distribution. For example, the Wilcoxon signed rank test is the non-parametric analog of the paired Student *t*-test, used in genomic investigations. Chi-square and Fisher exact tests compare frequencies of events. All tests are supported by *R*, and described in *R for Medicine and Biology*. *R for Medicine and Biology* also describes advanced R modules for multivariate analysis (correlation and regression in multiple dimensions), principle components analysis, clustering methods, survival analysis, and surveillance algorithms for epidemiologic outbreaks.

“Translational medicine” is the field-of-study that links results of basic research directly into patient–care applications, including diagnosis, prognosis, and therapy. In informatics applications, one requires connections and pipelines for highly heterogeneous data from different sources worldwide, in different formats, different languages, including continuous, categorical, text-based, and even de-identified/anonymized data for protection of patient privacy. *R for Medicine and Biology* discusses hash strings for encryption/decryption, creation of summary datasets, and export to heterogeneous target environments.

Later chapters in *R for Medicine and Biology* cover advanced topics, including tissue microarrays (TMAs) and gene expression analysis, using R in conjunction with the Bioconductor Project (open-source, academic software applications for genomic data). A “GeneChip array” is an array of many tiled areas, or cells, each with many copies of a unique tissue probe. Each tile, in turn, consists of smaller tiles of paired oligonucleotide probes, where each (pair) probe set contains a perfect match (PM) probe and a mismatch (MM) probe. Differences between PM and MM probes are tested for statistical significance using the Wilcoxon signed rank test. Statistically significant probes can be deposited in a publicly posted Gene Expression Omnibus (GEO) repository at the U. S. National Center for Biotechnical Information, as required by many U. S. funding agencies. *R for Medicine and Biology* shows how to query GEO subsets using the GEOquery package in R, with the example of polycystic ovary syndrome, including normalization, quality control, and annotation from the literature. Many human cancers exhibit genetic instability, in which one or a few nucleotides are substituted, lost, or gained, changing the protein structure encoded by that gene. Loss of heterogeneity (LOH) in a chromosome pair can lead to eventual inactivation of the protein encoded at that site, and inactivation of protein-based tumor suppression. Comparative genomic hybridization (CGH) is a fluorescence-based in-situ hybridization (FISH) technique for comprehensive screening of losses and gains across all chromosomes in a tumor sample, involving comparison of tumor and corresponding normal tissue from the same patient. Tumor DNA is labeled with green fluorochrome; corresponding normal DNA is labeled with red fluorochrome. Image analysis of human metaphase plates then localizes red/green ratios showing genetic loss or gain. *R for Medicine and Biology* describes the Manor package in R that normalizes array CGH data; and the Glad package in R that detects chromosomal breakpoints, assigning normal, gain, or loss status to these patients, with extensive graphical plots to normalize the results.

All text files beyond simple, raw text consist of the text itself, plus instructions for how the text should be displayed on a computer screen, paper printout, or file-sharing repository. These instructions are called “markup”. Hypertext Markup Language (HTML), which is the most widely-used markup language on the worldwide web, and serves as the “lingua franca” of the Internet. HTML manages paragraphing, font styles and sizes, colors, placement of images, etc., but does not address the “meaning” (semantics) of its contents. For example, a surgical pathology report with its accession number in the middle right margin of an HTML page is unreadable by software expecting the accession number on the top left margin of the HTML page.

Extensible Markup Language (XML) expands the functionality of markup in HTML to include the semantics of data objects. XML projects with special interest for pathology include the Foundational Model of Anatomy (FMA) and the Laboratory Digital Imaging Project (LDIP) of the Association for Pathology Informatics. Major granting agencies, such as the National Institutes of Health and the United Kingdom Medical Research Council, require data from grant-supported projects to be submitted for public viewing in XML format. Many leading biomedical journals likewise have XML data-sharing policies.

In a pathology report, XML markup might include patient identifiers, accession number, date–time when the specimen was obtained from the patient, date–time received in the laboratory, date–time electronically signed and released, etc. There are extensive features in XML that incorporate existing international standards, such as the date–time standard of the International Standards Organization, ISO 8601. These standards can resolve such paradoxical date–times as an outsourced laboratory result released electronically in South Bend, IN (Eastern Standard Time, EST), and reported to the provider apparently one hour earlier at a site in Gary, IN (Central Standard Time, CST). *R for Medicine and Biology* provides an introduction to the XML language, XML schemas, and use of the R editor to display, load, and parse an XML document, including data-mining, decision-tree searching, and encryption/decryption routines.

*R for Medicine and Biology* benefits greatly from the Appendix/Glossary at the end of the book. This feature, a hallmark of books in the Jones and Bartlett Biomedical Informatics series, encapsulates all the central ideas of statistical analysis and graphical display in an attractive, summary form. The reader is presented with the ideas of statistical estimation and hypothesis testing, with the commitment of only a few hours of focused reading. If these ideas seem interesting, then the reader can devote more time to the rest of the book.

No book can be all things to all readers. *R for Medicine and Biology* has the character of a do-it-yourself cookbook, written by a master chef statistical consultant, for the benefit of amateur consultants. In early chapters is the introductory section providing background and motivation, and the concluding summary section is short and perfunctory: if researcher X brings in a spreadsheet full of data to your office, then here is how you proceed. *R for Medicine and Biology* background sections for later chapters are more informative; but *R for Medicine and Biology* summary sections almost never really improve. Ideally, summary sections should recapitulate the issues raised in the initial background section and then recall how the issues have been resolved in the body of the chapter.

For example in Chapter 4, graphical display of data is not just a decoration, its a prerequisite and a necessity. The easy availability of powerful tools for statistical analysis should not seduce the investigator from simply looking at the points on various XY planes, and seeing what they say at a glance. Many utterly ridiculous or pointless hypotheses can be put to rest at the outset of an investigation with this simple approach. The author might even have presented some examples, such as the proverbial one-point high correlation: 999 points clustered around the XY origin, with a single, high-valued (*x, y*)-point (possibly even an erroneous data point), driving a deceptively significant statistical correlation coefficient.

It has been written that no pathologist truly controls the operation of his/her laboratory without access to all the individual reports of the department. In decades gone by, this control was exercised by retaining bound volumes of consecutive reports in the department, indexed with paper file cards, and available for review by all attending pathologists. Nowadays, the computer system manages consecutive case-numbering, verifies patient and provider identification, prints lists of delinquent and near-delinquent cases, and fetches prior reports for a given patient or diagnostic code. However, the days when a pathologist with a new management or research idea could simply pull a few volumes off the shelf, and test the idea for viability, are gone. Spreadsheet applications and statistical graphics programming packages such as R can return this control to the pathologist. All pathology informaticists, and many pathologists, can use spreadsheets at varying levels of sophistication. Every attending pathologist should have read-only access to case-by-case downloads of his/her own cases, on demand. Simple sorting operations on spreadsheet data can reveal outlier cases requiring urgent attention, and suggest management strategies for preventing recurrences in the future. Pathologist-informaticists should have the skills to collect and analyze their own data. *R for Medicine and Biology* provides the tools and methods that you can use to conduct these analyses.

Disclaimer: United States Government Work, uncopyrighted. This document does not necessarily represent the views or policies of any United States Government agency. This document is provided “as is”, without warranty of any kind, express or implied, including but not limited to the warranties of merchantability, fitness for a particular purpose and non-infringement. In no event shall the authors be liable for any claim, damages, or other liability, whether in an action of contract, tort or otherwise, arising from, out of, or in connection with the document or the use or other dealings made with the document.

